# The Effect of Sodium Hexametaphosphate on the Dispersion and Polishing Performance of Lanthanum–Cerium-Based Slurry

**DOI:** 10.3390/ma17194901

**Published:** 2024-10-06

**Authors:** Yan Mei, Wenjuan Chen, Xuean Chen

**Affiliations:** 1 College of Materials Science and Engineering, Beijing University of Technology, Beijing 100124, China; 2 Key Laboratory of Advanced Functional Materials, Ministry of Education of China, College of Materials Science and Engineering, Beijing University of Technology, Beijing 100124, China

**Keywords:** abrasive, ceria, sodium hexametaphosphate, dispersion, chemical mechanical polishing

## Abstract

A lanthanum–cerium-based abrasive composed of CeO_2_, LaOF, and LaF_3_ was commercially obtained. The effect of sodium hexametaphosphate (SHMP) on powder dispersion behavior was systematically investigated using the combined techniques of liquid contact angle, turbidity, zeta potential (ZP), scanning electron microscopy (SEM), powder X-ray diffraction (XRD) combined with Rietveld refinements, X-ray photoelectron spectroscopy (XPS), and polishing tests. The results indicated that the addition of 0.5 wt.% SHMP dispersant to the 5 wt.% lanthanum–cerium-based slurry produced the most stable suspension with a high turbidity of 2715 NTU and a low wetting angle of 45°. The as-obtained slurry displayed good surface polishing quality for K9 glass, with low surface roughness (Ra) of 0.642 and 0.515 nm (in the range of 979 × 979 μm^2^) at pH = 6 and 11, respectively, which corresponds to the fact that it has local maximum absolute values of ZP at these two pH values. SEM images demonstrated that after appropriate grafting of SHMP, the particle aggregation was reduced and the slurry’s dispersion stability was improved. In addition, the dispersion mechanism was explained based on the principle of complexation reaction, which reveals that the dispersant SHMP can increase the interparticle steric hindrance and electrostatic repulsions. In an acidic environment, steric hindrance dominates, while electrostatic repulsion prevails under alkaline conditions. As expected, this polishing slurry may find potential applications in manufacturing optical devices and integrated circuits.

## 1. Introduction

Chemical mechanical polishing (CMP) is an important technique used to achieve nanoscale global planarization, and it has been widely used in precision manufacturing fields such as optical devices and integrated circuits (IC) for decades. CMP can achieve local and overall flatness of the wafer surface by removing materials at the micrometer, nanometer, or atomic level [1,2]. The principle of CMP is to remove materials via the mechanical wear of polishing particles and chemical corrosion in polishing slurry [3,4]. It combines the advantages of both mechanical and chemical polishing techniques, achieving chemical–mechanical synergy, which can improve the material removal rate (MRR) and surface quality of workpieces, thereby obtaining smooth and non-destructive polished surfaces [5,6,7].

Polishing slurry is one of the key elements in the CMP process. It plays a crucial role in polished workpieces’ MRR and surface roughness. Polishing slurry is a stable mixture of dispersed polishing particles (abrasive) and other chemical substances (such as oxidants, inhibitors, surfactants, and acid–base regulators) [8,9]. Commonly used abrasives mainly include SiO_2_, Al_2_O_3_, CeO_2_, etc., among which CeO_2_ has aroused great interest due to its variable valence state, high reactivity, slightly lower hardness than SiO_2_, and strong affinity to SiO_2_, and is widely used in IC polishing [1,10]. The Ce^3+^ concentration, morphology, and size of polishing particles are the decisive factors affecting the performance of CeO_2_ polishing slurry. From the perspective of chemical reactions in the CMP process, Ce^3+^ in the CeO_2_ abrasive reacts with the SiO_2_ surface to form the Ce–O–Si bond, thereby improving MRR and polishing efficiency [1,11]. The higher the concentration of Ce^3+^ is, the better is the polishing performance of CeO_2_ abrasive [11]. From the perspective of the mechanical removal effect during the CMP process, the morphology and size of polishing particles are also key factors affecting MRR. Although non-spherical abrasives generally have higher MRRs, current CMP techniques prefer spherical and smaller particle-size CeO_2_ abrasives because they can reduce scratches and surface roughness on polished parts [12].

It is well known that CeO_2_ ultrafine polished particles are prone to aggregation due to their size and surface effects. Therefore, in industrial production applications, to improve the polishing efficiency and surface quality of the CMP process, it is usually necessary to add surfactants (dispersants) to the polishing slurry to suppress particle agglomeration. The main factors affecting the dispersion stability of particles in polishing slurry are the type, the amount of dispersant added, and the pH value of the suspension [13,14]. Among various candidates, sodium hexametaphosphate (SHMP) is currently the most commonly used dispersant for polishing slurry [15]. It is a long-chain inorganic phosphate with the molecular formula (NaPO_3_)_6_. In an aqueous solution, the P–O groups are further interconnected via corner-sharing to form long-chain polymers, i.e., (NaPO_3_)_n_, *n* = 20–100 [16]. The spiral long-chain polymer anions formed by the polymerization of phosphate anions will create complexation reactions with non-alkali-metal cations on the surface of mineral particles, which increases the thickness of the hydration layer on the mineral surface and the negative potential of the mineral surface, thereby enhancing the repulsive force between mineral particles [17].

Several researchers have proposed different views on the dispersion mechanism of SHMP. For example, Wei et al. [18] found that SHMP can increase the mutual repulsion between particles by forming steric hindrance between them and increasing the zeta potential of the particle surface. This can overcome van der Waals forces to a certain extent, reduce the aggregation of the nanosized ceria particles, and thus improve the dispersion stability of particles in the slurry. Austin et al. [19] studied the role of SHMP in wet-stirred milling of Al-doped TiO_2_. They found that SHMP strongly interacts with Al-doped TiO_2_ in both acidic and basic conditions, stabilizing the particles via steric and electrostatic forces. Luo et al. [20] investigated the dispersion behavior of submicron cerium oxide particles in concentrated suspensions using an ultrasonic attenuation technique. They pointed out that for suspensions with concentrations ranging from 10 wt.% to 30 wt.%, the ceria particles are most sufficiently dispersed in suspensions containing SHMP. SHMP can increase the zeta potential of ceria submicron particle surfaces in the slurry and enhance the electrostatic stability of the particles. Liao et al. [21] examined the effects of SHMP on the flotation separation of fine smithsonite from calcite and its mechanism. Their results demonstrated that SHMP acted as a dispersant to significantly decrease the zeta potential of the mineral particles, which led to the improvement of interparticle repulsion and full dispersion of the fine smithsonite and calcite powders. Yang et al. [22] investigated the effects of the addition of dispersant SHMP and the pH value of the suspension on the suspension performance of ceria. The results indicated that PO_3_^−^ of SHMP can coordinate with Ce^3+^ on the surface of CeO_2_, and high pH can promote this coordination reaction, which increases the absolute value of the zeta potential of particles and improves the dispersion stability of CeO_2_ particles in solution. Han et al. [15] elucidated the impact of SHMP on the dispersion performance of the micro-sized lanthanum–cerium-based slurry. They pointed out that the dispersion mechanism of SHMP is the combination of steric hindrance and electrostatic repulsion when the pH of the slurry is smaller than the isoelectric point. At higher pH values, electrostatic repulsion was dominant.

Although many studies have been conducted on the dispersion mechanism of SHMP in different suspension systems, the findings reported in the literature are controversial. In addition, a systematic investigation of the effect of adding SHMP dispersant on the dispersion behavior and polishing performance of lanthanum–cerium-based abrasive is still limited. In a previous study, we reported a lanthanum–cerium–fluoride abrasive and investigated the influence of calcination temperature on its phase composition, morphology, surface elemental oxidation state, and polishing performance [23]. As a continuation of our previous research, in this study, we investigated the effect of adding SHMP dispersant on the wettability, suspension, sedimentation rate, particle morphology, phase composition, and Ce^3+^ surface concentration of this lanthanum–cerium-based polishing slurry, and determined the optimal amount of SHMP added. In particular, the phase composition of this system obtained from Rietveld refinements has not been reported in the literature. In addition, the CMP polishing effect was verified using K9 glass, and the dispersion mechanism of SHMP in polishing slurries was explained based on the principle of complexation reaction. We believe this work will contribute to developing new abrasives for polishing optical glass surfaces.

## 2. Materials and Methods

### 2.1. Materials Synthesis

The starting material used in this study, lanthanum–cerium-based abrasive, is a commercial abrasive provided by Grinm Advanced Materials Co., Ltd. (Beijing, China). According to the analysis results of inductively coupled plasma optical emission spectroscopy, it consists mainly of 93.4 wt.% rare-earth oxide (63.89 wt.% CeO_2_ + 36.11 wt.% La_2_O_3_) and 5.22 wt.% fluorine. The particle size ranges from 600 to 800 nanometers. The dispersant, sodium hexametaphosphate [SHMP, (NaPO_3_)_6_, molecular weight = 611.77, degree of polymerization = 6], is of chemical grade and was purchased from Shandong Jingze Optical Material Co., Ltd. (Zibo, China). The acid–base regulators HCl and NaOH are analytical reagents obtained from Fuchen Chemical Reagent Co., Ltd. (Tianjin, China). Deionized water obtained through a water purification system (Beijing Aisitaike Technology Development Co., Ltd., Beijing, China) was used throughout the experiment. All chemicals were used directly without further purification.

The polishing slurries, composed of 5 wt.% lanthanum–cerium-based abrasives with the addition of 0%, 0.3%, 0.4%, 0.5%, 0.6%, 0.8%, 1.5%, and 3.0% (in weight percentage, wt.%) SHMP dispersant, were prepared according to the following procedure. First, the calculated amounts of lanthanum–cerium-based abrasive, SHMP, and deionized water were weighed (or measured) and mixed in a 500 mL beaker. The mixture was continuously stirred with a fixed speed of 600 r·min^−1^ on a magnetic stirrer (78–1, Jintan Medical Instrument Factory, Changzhou, China) for 2 hours to ensure that the system reached a balance. Subsequently, the pH value of the obtained slurry was adjusted to 3–12 with HCl and NaOH for subsequent investigations of wettability, suspension, zeta potential, and polishing properties. Meanwhile, the selected lanthanum–cerium-based slurries with and without the addition of SHMP were centrifuged to obtain solid particles, which were dried and then collected for further SEM, powder X-ray diffraction (XRD), and X-ray photoelectron spectroscopy (XPS) characterization.

For the measurement of zeta potential, 10 clean test tubes and 10 pH adjustment solutions of HCl and NaOH with pH values of 3–12 were first prepared. Then, a 1 mL polishing solution was transferred to each test tube via a pipette. Based on the initial pH of the polishing solution and the change in pH after adding an acid–base regulator, suitable pH regulators were selected and dropped into each test tube using a dropper to adjust the pH of the polishing solution to 3–12, thus obtaining 10 samples. The total volume of the polishing solution in each test tube was maintained at 10 mL so that it was diluted 10 times relative to the original polishing solution. This solution was subsequently used for zeta potential measurements, for which each sample was tested three times, with a 10 s interval between each test. Finally, three zeta potential values were determined for each sample and the average value was used for the analysis.

### 2.2. Material Characterization

The chemical composition of the starting abrasive was measured by inductively coupled plasma optical emission spectroscopy (ICP-OES, Optima 7000 DV, Perkin-Elmer, Waltham, MA, USA). The pH value of the suspension was determined by an online glass electrode pH-meter (SD20 Kit, Mettler Toledo, Bedford, MA, USA) with a measurement accuracy of ±0.002. The contact angle (wetting angle) between the suspension and the glass surface was measured using a surface tension/contact angle meter equipped with a digital camera (GS series, SurfaceTech. Co., Ltd., Gwangju, Republic of Korea). The slurry was centrifuged by a high-speed centrifuge (TDL80-2B, Shanghai Anting Scientific Instrument Co., Ltd., Shanghai, China). The turbidity of the supernatant after centrifugation was measured with a turbidimeter (TL2300, Hach Company, Loveland, CO, USA). The zeta potential of the particles in the slurry was evaluated by a nanoparticle size and zeta potential analyzer (NiCOMP Z3000, PSS, Santa Barbara, CA, USA). Microstructure and surface morphology of the polished particles were observed using a high-resolution cold field emission scanning electron microscope (SEM, HELIOS 600i, FEI Company, Hillsboro, OR, USA) at an acceleration voltage of 10.0 kV. Powder X-ray diffraction (XRD) patterns of the abrasives were recorded at ambient temperature in a continuous scan mode on a Bruker D8 ADVANCE (Bruker AXS, Karlsruhe, Germany) diffractometer using the monochromatized Cu K_α_ radiation (*λ* = 1.5406 Å). The operating voltage and current of the X-ray tube were fixed at 40 kV and 40 mA, respectively. Intensity data were collected from 2θ = 10 to 100°, with a step size of 0.02° and a step time of 10 s. X-ray photoelectron spectroscopy (XPS, ESCALAB 250xi, Thermo Fisher Scientific, Waltham, MA, USA) was used to determine the surface chemical composition of polished particles. The spectrum was obtained by focusing monochromatized Al K_α_ radiation (hν = 1486.6 eV) and calibrated by applying an exogenous C 1s signal at 284.6 eV. Quantitative analysis of the compact surface roughness of polished glass was performed using an optical profilometer, which was interfaced with MetroPro™ imaging software (NewView 5000^TM^, Zygo Corporation, Middlefield, CT, USA), with measurements taken over an area of 979 × 979 μm^2^ with a 10× objective lens.

## 3. Results and Discussion

### 3.1. Effect of SHMP on Wettability and Suspension of the Slurry

**Figure 1:**
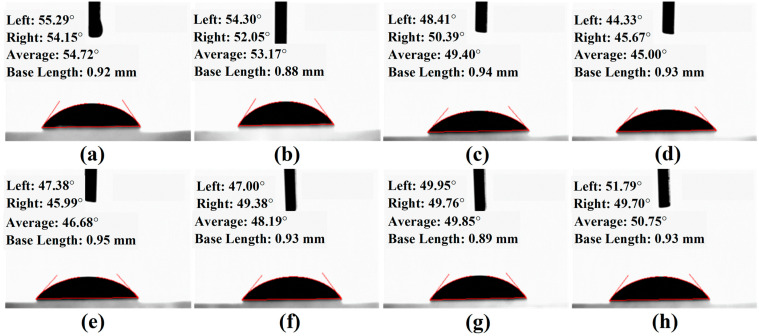
Liquid contact angles of the polishing slurries consisting of 5% lanthanum–cerium-based abrasives with the addition of 0% (**a**), 0.3% (**b**), 0.4% (**c**), 0.5% (**d**), 0.6% (**e**), 0.8% (**f**), 1.5% (**g**), and 3.0% (**h**) SHMP dispersants, respectively.

Cerium-based polishing powder has the characteristics of small particle size, large specific surface area, and high specific surface energy. It is prone to aggregation and sedimentation during storage and application, seriously affecting its performance. In order to suppress agglomeration, the polishing powder is usually dispersed in a medium (water or organic solvent) to form a highly dispersed and stable polishing solution. Therefore, its performance can be assessed based on some of the slurry’s liquid properties, with two commonly used evaluation indicators being wettability and suspension. Wettability refers to a liquid’s ability and tendency to spread on a solid’s surface. For polishing slurry, good wettability means that the polishing liquid spreads evenly and tightly on the surface of the workpiece to be polished, allowing more polishing particles to come into contact with the workpiece surface, thereby improving the polishing speed and efficiency [24,25]. The liquid contact angle is a parameter used to measure the wettability of a liquid on a solid surface. Its value depends on the characteristics of the liquid and solid surfaces, and a smaller contact angle indicates good interfacial wettability [24,26]. The liquid contact angles of the lanthanum–cerium-based slurries with different concentrations of SHMP added were measured, as shown in Figure 1.

**Figure 2:**
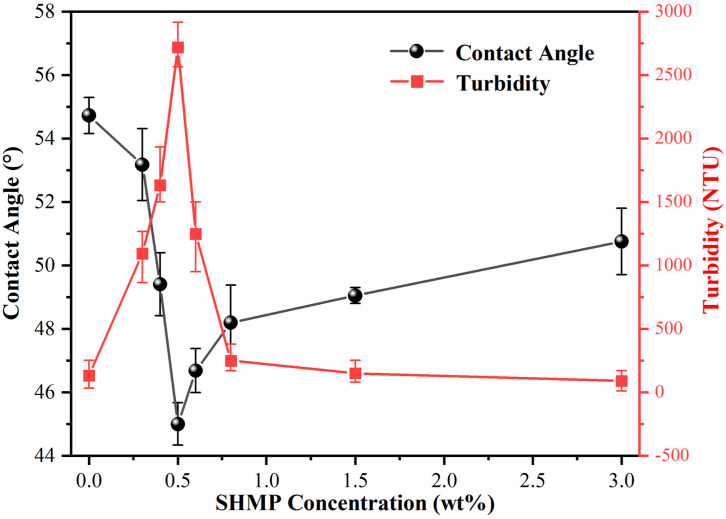
Variation in contact angle and turbidity as a function of the SHMP concentration.

A plot of the contact angle as a function of the SHMP addition amount is displayed in Figure 2. It was found that the contact angle of the polishing slurry consisting of 5% lanthanum–cerium-based abrasive on the glass was 54.72°. It decreased rapidly as the SHMP concentration increased from 0% to 0.5%, indicating a significant improvement in the wetting performance of the polishing solution on the glass substrate. However, once the amount of SHMP was further increased to above 0.5%, the contact angle increased, first quickly and then slowly, showing that excessive SHMP is unfavorable for the chemical reaction at the glass–slurry interface.

Suspension refers to the ability of particles to maintain a suspended and stable state in a dispersed system, typically evaluated by turbidity values. High turbidity reflects the dispersion of the particles in the suspension, while low turbidity means that most particles are sedimented to the bottom of the suspension [27]. As mentioned above, CeO_2_ particles exhibit a strong tendency to aggregate due to their high surface polarity, high tension, and hydrophobic nature, which leads to poor suspension stability of the slurry, thus affecting the surface quality of polished products [28]. To investigate the suspension of the system, the 5% lanthanum–cerium-based polishing slurries doped with different amounts of SHMP were first centrifuged. Then, the turbidity of the as-obtained supernatants was measured, as shown in Figure 2. It could be observed that the turbidity of the slurry in the absence of SHMP was very low, indicating poor suspension performance. Furthermore, the turbidity rose rapidly as the SHMP amount increased from 0.3% to 0.5%. It reached a maximum of 2715 NTU when the SHMP concentration was 0.5%, indicating that the CeO_2_ particles are well dispersed in the aqueous medium, and aggregation and sedimentation are significantly suppressed. Upon further increasing SHMP concentration from 0.6 to 0.8%, the turbidity of the solution decreased significantly, indicating that the particles tend to deposit at the bottom of the suspension, weakening the stability of the suspension. When the amount of SHMP increased to above 0.8%, the turbidity continued to decrease, but at a slower rate. After the SHMP content exceeded 3.0%, the turbidity was even lower than that of the original slurry without dispersants, which means that the excessive addition of SHMP dispersant will weaken the suspension stability of the slurry.

**Figure 3:**
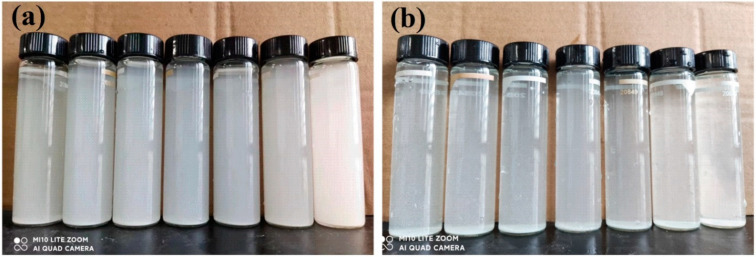
Photos of the supernatants of lanthanum–cerium-based slurries with different SHMP additions after centrifugation (**a**) and after centrifugation followed by settling for 4 h (**b**). From left to right, the addition amounts of SHMP are 0.3%, 0.4%, 0.5%, 0.6%, 0.8%, 1.5%, and 3.0%, respectively.

**Figure 4:**
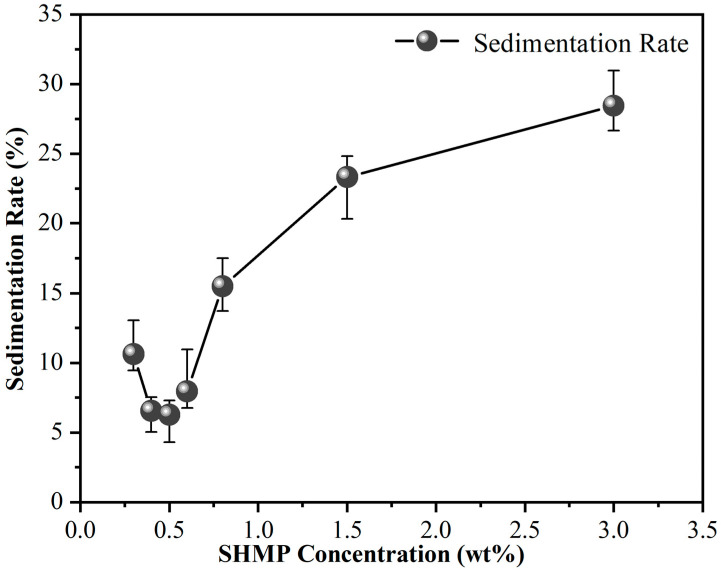
Variation in the sedimentation rate of the lanthanum–cerium-based polishing slurry as a function of the SHMP addition amount.

To more clearly describe the dispersion stability of the slurry, the following stability tests were conducted: 0.3%, 0.4%, 0.5%, 0.6%, 0.8%, 1.5%, and 3.0% SHMP dispersants were first added to the 5% lanthanum–cerium-based polishing slurries, respectively. Subsequently, the slurries were centrifuged, and the obtained supernatants were left to stand in test tubes for 4 h, as shown in Figure 3. After that, referring to the method adopted by Yang et al. [22], the heights of the supernatant layer and the total solution layer in the test tube were measured. Their percentages were defined as sedimentation rate, another commonly used indicator for evaluating suspension. The sedimentation rate is inversely proportional to the suspension stability of the dispersion system, i.e., the lower the sedimentation rate is, the better the diversification effect will be [29]. The impact of adding SHMP on the sedimentation rate of polishing slurry is shown in Figure 4. When the amount of SHMP was less than 0.5%, the sedimentation rate decreased with the addition of SHMP. This means that an appropriate amount of SHMP helps to disperse CeO_2_ particles into the aqueous medium, thereby improving the suspension stability of the polishing solution. When the SHMP concentration was greater than 0.5%, the sedimentation rate increased steadily with the dispersant content, indicating a deterioration of the suspension stability of the slurry. The above results indicated that with the addition of SHMP, the liquid contact angle and sedimentation rate of the slurry first declined and then increased, while the turbidity showed the opposite trend. This is because the proper grafting of SHMP provides more hydrophilic P–O groups, enhancing the hydrogen bond between the abrasive and the glass substrate. It also provides more reaction sites for the chemical reactions between the abrasive and the glass and increases the contact area of the mechanical action. However, the excessive addition of SHMP will lead to bridging flocculation due to the cross-bonding of (PO_3_)_n_^n−^ polymeric chains [30,31], weakening the stability of the suspension. The optimal doping concentration for SHMP is 0.5%.

The above results can be explained according to the complexation reaction principle. SHMP consists of Na^+^ ions and (PO_3_)_n_^n−^ groups, of which Na^+^ can undergo ion exchange reactions with Ce^3+^ and Ce^4+^ in the ceria-based abrasive. Additionally, the P=O double bond in the (PO_3_)_n_^n−^ group has a strong van der Waals attraction to Ce^3+^ ions, which enables (PO_3_)_n_^n−^ to adsorb on the surface of ceria particles to make complexation reactions with Ce^3+^. This can increase the thickness of the double electrical layer on the surface of ceria particles and cause steric hindrance between particles, which physically prevents the particles from approaching close enough to cause agglomeration, thereby enhancing the suspension performance of the slurry. Moreover, (PO_3_)_n_^n−^ can also help to improve the hydrophilicity of the surface of ceria particles and enhance the wettability of the slurry on the glass substrate. This can increase the contact area during the CMP process, which benefits the mechanical effect [24]. Ding et al. [32] found that the MRR positively correlates with the number of effective particles and the contact area of abrasive particles. When polishing with a slurry composed of 5% lanthanum–cerium-based abrasive doped with 0.5% SHMP, the steric hindrance effect of the long-chain (PO_3_)_n_^n−^ groups allows the abrasive particles to be uniformly dispersed, leading to an increase in the number of effective particles involved in the polishing process. On the other hand, adding an appropriate amount of SHMP may reduce the slurry’s contact angle on the glass surface. This means that a larger contact area can be generated between the abrasive and the glass substrate, and the particles are more easily absorbed on the glass surface. Combining these two aspects brings about a superior polishing performance of the slurry. However, excessive SHMP can cause surplus (PO_3_)_n_^n−^ to exist in the medium. These polymeric chains can be interconnected to form a network structure, resulting in bridging flocculation and suspension instability. Therefore, the optimal amount of SHMP added to the 5% lanthanum–cerium-based polishing solution for achieving the best dispersion effect is about 0.5%. The as-obtained polishing solution has a high turbidity of 2715 NTU and a low wetting angle of 45°.

### 3.2. Effect of pH Value on Zeta Potential of the Slurry

**Figure 5:**
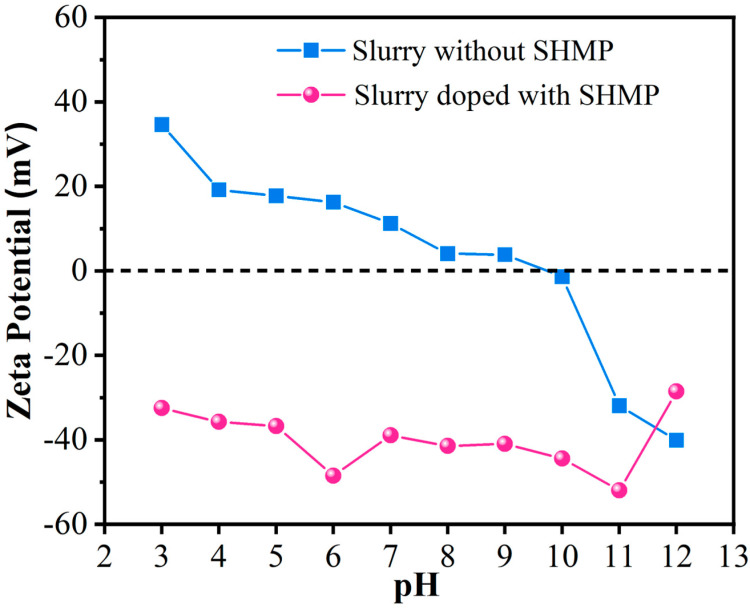
Plots of zeta potential of the polishing slurries consisting of 5% lanthanum–cerium-based abrasives doped with 0.5% SHMP and without SHMP as a function of pH.

Zeta potential (ZP) is a physical quantity that characterizes the charge state of particles in a slurry. It represents the potential stability of the suspension and indicates the degree of repulsion between adjacent, similarly charged particles in the dispersing medium. It is strongly affected by the pH value of the solution. The higher the absolute value of ZP is, the greater is the electrostatic repulsion between particles, and the more stable the suspension can be [27,33]. To further investigate the stability of the suspension, the charge state of particles in the polishing solution, and the suitable acid–base environment of the polishing solution, we chose a typical 5% lanthanum–cerium-based polishing slurry doped with 0.5% SHMP as an example to investigate the influence of pH on the ZP. Figure 5 shows the ZP values of this polishing slurry recorded over a wide pH range of 3–12 compared to those of the corresponding slurry without SHMP.

It can be observed that when no dispersant was added, the ZP value of the lanthanum–cerium-based slurry was positive (34.65 mV) at pH = 3, which might be due to the protonation of surface hydroxyl groups (Equation (1), [15,34]):

Ce-OH + H^+^ ↔ Ce-OH_2_^+^ (1)

As the pH value increased, the ZP value of the slurry gradually decreased. When the pH value was greater than or equal to 10, the ZP value became negative due to the deprotonation of surface hydroxyls (Equation (2), [15,34]):

Ce-OH ↔ Ce-O^−^ + H^+^ (2)

In the absence of SHMP, the isoelectric point (IEP) of the lanthanum–cerium-based slurry lies between pH = 9 and 10, which is different from that reported in Ref. [15] (IEP pH = 8.3), possibly due to the different phase compositions of these two slurries (CeO_2_ + LaOF + LaF_3_ in this work vs. CeO_2_ + LaOF in Ref. [15]).

When 0.5% SHMP was added to a 5% lanthanum–cerium-based polishing solution, it was found that the ZP of the polishing solution became negative and its absolute value mostly increased sharply. This phenomenon occurs because SHMP, an anionic dispersant, adsorbs onto the surface of the abrasive, thereby improving the negative charge density. Similar ZP changes have also been previously reported [15,35]. As the pH value varied from 3 to 6, in the presence of SHMP, the ZP changed from −32.50 to −48.49 mV, which means that under acidic to weakly acidic environments, an increase in pH value will improve the dispersion stability of the CeO_2_ particles in the polishing solution. When the solution was nearly neutral (pH~7), the absolute value of ZP dropped significantly. In this case, the electrostatic repulsion between the surfaces of CeO_2_ particles was the weakest, and the dispersion performance of the slurry was the worst. When the solution was alkaline (pH = 9–11), the absolute value of ZP showed an increasing tendency again along with increasing pH, and its maximum value (−51.99 mV) was located at pH = 11. However, once the pH value exceeded 11, the absolute value of ZP rapidly decreased, suggesting that a strong alkaline environment would weaken the suspension performance of the polishing solution. Generally speaking, a ZP of 30 mV (positive or negative) can be taken as the critical value for judging the dispersion stability of particles [36]. For the abrasive treated with SHMP, at pH = 12, the ZP was −28.55 mV, with an absolute value being less than 30 mV. This means that in this situation, the electrostatic repulsion is insufficient to overcome the van der Waals attraction between abrasives, making it difficult to maintain suspension stability. Moreover, the abrasive used in this study is mainly composed of CeO_2_, with some admixtures of LaOF and LaF_3_. At room temperature, cerium dioxide is insoluble in water and alkali, but slightly soluble in acid, and its solubility increases sharply with the decrease in pH. Therefore, this material can be used as an abrasive for polishing glass under nearly neutral and alkaline conditions.

Hydrated CeO_2_ exhibits acid–base duality: in acidic environments, hydroxyl protonation occurs on the surface of CeO_2_ to generate –Ce–OH_2_^+^ [Equation (1)], while under alkaline conditions, the hydroxyl on the surface of CeO_2_ is deprotonated to form Ce–O^−^ [Equation (2)] [15,34]. Moreover, SHMP is a glassy polyphosphate and long-chain inorganic salt with the formula (NaPO_3_)_n_. It is a spiral chain polymer derived via polymerization of numerous PO_4_^3−^ basic structural units, with a polymerization degree (*n*) of about 20–100 [16]. When SHMP is in an aqueous medium, it will be hydrolyzed and converted into long chains with negative charges [37]. When SHMP is added to the ceria-based polishing slurry in acidic environments, the negatively charged (PO_3_)_n_^n−^ and positively charged –Ce–OH_2_^+^ will generate electrostatic attraction to neutralize the positive and negative charges, which will prevent direct particle–particle contact due to the formation of a significant steric barrier between them, thus enhancing the dispersion stability of the CeO_2_ slurry. On the other hand, when SHMP is added under alkaline conditions, it is difficult for negatively charged (PO_3_)_n_^n−^ to adsorb on the particle surface of negatively charged Ce–O^−^ due to electrostatic repulsion. These increases the ZP of particle surfaces, which can overcome van der Waals attraction between ceria particles, prevent particles from agglomeration, and thus, improve the dispersion stability of CeO_2_ particles in the slurry. The fact that the maximum absolute value of ZP was observed at pH = 11 confirms this point. Based on the above analysis, there are two possible explanations for the dispersion mechanism of ceria particles with SHMP. Firstly, there is a large amount of strongly negatively charged (PO_3_)_n_^n−^ groups in the long-chain structure of SHMP, which enhances the electrostatic repulsions between particles. Secondly, the adsorption of an appropriate amount of SHMP on the surface of ceria particles increases the spatial steric hindrance between particles and effectively alleviates polymerization [37]. Since hydrated CeO_2_ may exist in the form of –Ce–OH_2_^+^ or Ce–O^−^, depending on the pH value of the solution, we believe that in acidic environments, the negatively charged (PO_3_)_n_^n−^ can easily be adsorbed to the surface of positively charged –Ce–OH_2_^+^ particles, enter the diffusion layer, and form Ce^3+^–(PO_3_)_n_^n−^ complexes; therefore, the steric hindrance is dominant. In contrast, it is difficult to combine negatively charged (PO_3_)_n_^n−^ with Ce–O^−^ groups into complexes under alkaline conditions, so the electrostatic repulsion prevails. These conclusions support the argument put forward by Han et al. [15].

### 3.3. Effect of SHMP on the Morphology of Abrasives

**Figure 6:**
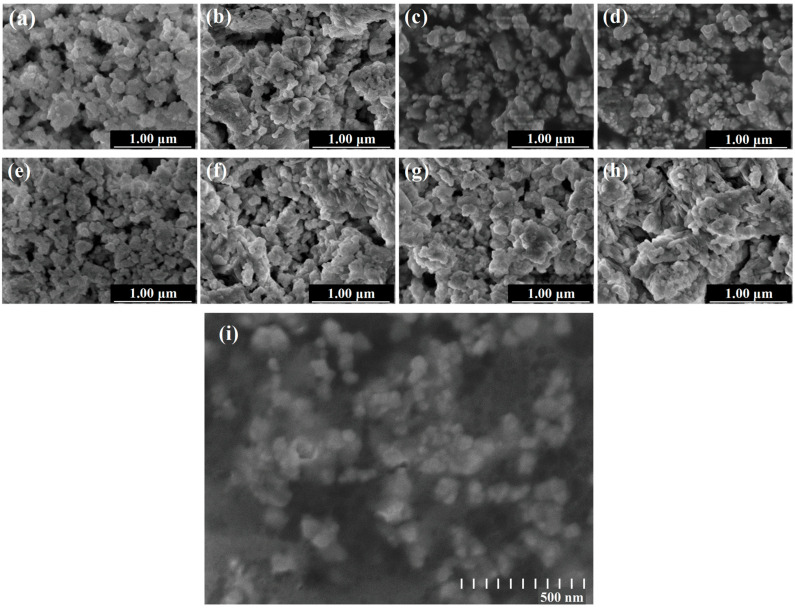
SEM images of 5% lanthanum–cerium-based polishing slurries with the addition of 0% (**a**), 0.3% (**b**), 0.4% (**c**), 0.5% (**d**), 0.6% (**e**), 0.8% (**f**), 1.5% (**g**), and 3.0% (**h**) SHMP dispersant, respectively, along with a typical magnified micrograph of 5% lanthanum–cerium-based abrasive doped with 0.5% SHMP (**i**).

The particle morphology of abrasives is an essential factor affecting the polishing quality of workpieces. To study the microstructure and surface morphology of polished particles, lanthanum–cerium-based slurries doped with different concentrations of SHMP were centrifuged and dried. SEM images of the obtained solid particles were taken, as shown in Figure 6, where the change in particle morphology and size caused by the addition of SHMP can be clearly observed. Overall, the material is agglomerated and has an irregular block-like morphology with a broad particle size distribution ranging from tens to hundreds of nanometers. Furthermore, as the SHMP-doped content increased from 0% to 0.5%, the morphology of the polishing material gradually changed from many clumps to many small particles with visible boundaries. A typical magnified micrograph of a 5% lanthanum–cerium-based abrasive doped with 0.5% SHMP is also provided in Figure 6, which shows that aggregation was suppressed and the particle size was in the range of about 50–100 nanometers. This is because the long-chain structure of SHMP provides a strong steric hindrance effect on abrasive particles, which physically prevents the particles from approaching enough to cause agglomeration. In addition, the water solubility of the (PO_3_)_n_^n−^ group in SHMP makes abrasive particles hydrophilic and soluble in aqueous medium. The combination of these two aspects leads to an improvement in the dispersion stability of the slurry. As a result, the hard damage caused by abrasive agglomeration will be reduced, and the surface quality of the workpiece after chemical mechanical polishing will be improved. Upon further increasing the SHMP concentration from 0.6 to 3.0%, the granular structure gradually became clump-like again. In this case, particles tend to re-agglomerate due to the bridging flocculation mentioned above, and a weakening of the stability of the suspension is expected. All of these observations are consistent with the variation trends in the turbidity and wetting angle of the polishing slurries in Figure 2 and conform to the theoretical analysis of the complexation reaction principle.

### 3.4. Effect of SHMP on the Phase Purity of Abrasives

**Figure 7:**
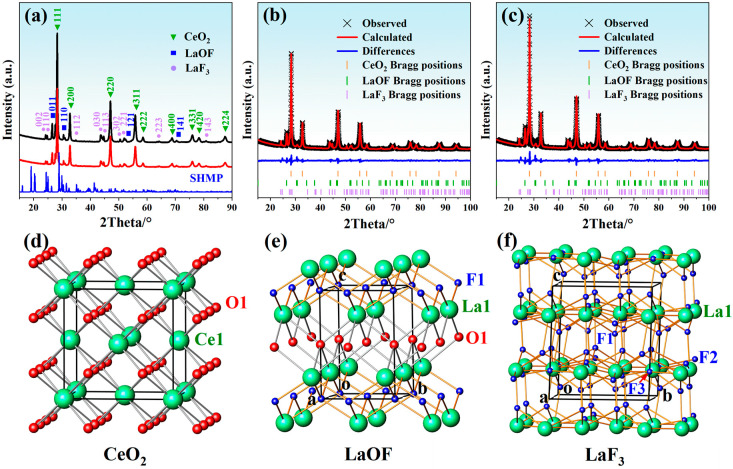
(**a**) XRD patterns of lanthanum–cerium-based abrasives without (middle) and with the addition of SHMP (top) together with that of SHMP (bottom, simulated from ICSD#34693 for (NaPO_3_)_n_) for comparison. Rietveld plots of lanthanum–cerium-based abrasives without (**b**) and with the addition of SHMP (**c**). Also shown are the crystal structures of CeO_2_ (**d**), LaOF (**e**), and LaF_3_ (**f**).

To investigate the effect of adding SHMP on the phase purity of abrasives, the polishing slurries consisting of 5% lanthanum–cerium-based abrasives doped with 0.5% SHMP and without SHMP were centrifuged and dried, and analyzed by XRD, as shown in Figure 7a. The diffractogram showed strong peaks at 2θ = 28.31°, 32.79°, 47.07°, 55.83°, 58.54°, 68.82°, 76.00°, 78.32°, and 87.46°, corresponding to the crystal planes (111), (200), (220), (311), (222), (400), (331), (420), and (224) of the cubic ceria phase (PDF#34–0394). Moreover, several other peaks were also observed at 2θ values of 26.54°, 30.53°, 52.38°, and 70.41°, which belong to the (011), (110), (121), and (141) planes of the tetragonal LaOF phase (PDF#89–5168). The remaining diffraction peaks were ascribed to the reflection of trigonal LaF_3_ (PDF#84–0942). The XRD pattern confirmed the coexistence of CeO_2_, LaOF, and LaF_3_ in the abrasive. Adding SHMP to the slurry did not introduce a secondary phase, except for a slight change in diffraction peak intensity.

To further understand the crystal structure and phase composition of abrasives, Rietveld refinements were performed on the powder XRD data using TOPAS software [38]. During the refinement, the modified Thompson–Cox–Hastings pseudo-Voigt function was used to simulate the peak profile [39], the Chebyschev polynomial function was used to describe the background, and the spherical harmonic method was used to model the preferred orientation. The initial structural models were taken from the Inorganic Crystal Structure Database (ICSD#621716 for CeO_2_, ICSD#76427 for LaOF, and ICSD#201865 for LaF_3_) [40,41,42], where the positions of heavy atoms (La in both LaOF and LaF_3_) were refined. In contrast, the positions of the other atoms were fixed. The observed and calculated XRD profiles, their difference curves, and the Bragg positions of two typical samples are shown in Figure 7b,c. Detailed crystallographic data and fitting parameters are listed in Table 1. Refinements produced satisfactory profile factors (R_p_ < 5%, R_wp_ < 7%, and GOF < 4), again confirming that the abrasives are a mixture of cubic CeO_2_, tetragonal LaOF, and trigonal LaF_3_, which crystallize in the space groups *Fm*-3*m*, *P*4/*nmm*, and *P*-3*c*1, respectively, with lattice constants consistent with those reported in references [40,41,42]. Adding SHMP dispersant to the abrasive did not produce any distinct impurities or induce significant changes in the crystal structure, except for minor variations in phase fraction, as shown in Table 1. It is worth noting that in a previous study [15], the XRD pattern of the lanthanum–cerium-based abrasive treated with SHMP showed not only characteristic peaks of CeO_2_ and LaOF, but also a strong peak at 2θ = 14.46°, which matches well with CePO_4_ (JCPDS No. 01-074-1889). This suggests that adding SHMP can cause a certain amount of Ce^3+^ on the abrasive surface to combine with PO_4_^3−^ of SHMP to form a new phase. However, in this work, the XRD pattern of the lanthanum–cerium-based abrasive treated with SHMP is almost identical to that of the abrasive without SHMP. This implies that adding SHMP can only help disperse CeO_2_ particles into the aqueous medium, without causing the formation of new phases. Of course, there is another possibility, that is, Ce^3+^ on the surface of the abrasive reacts with PO_4_^3−^ of SHMP to form cerium phosphates, which crystallize into amorphous phases with relatively low phase content and cannot be detected by XRD.

The CeO_2_, LaOF, and LaF_3_ crystal structures are also presented in Figure 7d–f. Among them, CeO_2_ has a fluorite-type cubic structure with a space group of *Fm*-3*m* and a point-group symmetry of *O_h_*. In this structure, Ce^4+^ cations form a face-centered cubic (*f.c.c.*) array, and O^2−^ anions occupy all tetrahedral interstitial sites. Each Ce^4+^ is bound to eight O^2−^ anions to form a CeO_8_ cube, while each O^2−^ is surrounded by four Ce^4+^ ions to constitute a distorted O-centered tetrahedron. LaOF crystallizes in the tetragonal space group of *P*4/*nmm* (D_4h_^7^, No. 129). It has a layered structure consisting of alternating O^2−^ and F^−^ anionic layers arranged perpendicular to the *c*-axis, with La^3+^ cations located in the square prismatic cavities between the layers. La^3+^ ions are eight-fold coordinated, bound to four O^2−^ and four F^−^ anions, while both O^2−^ and F^−^ ions are tetrahedrally coordinated to La^3+^ ions. LaF_3_ possesses the trigonal tysonite structure, which belongs to the *P*-3*c*1 space group, with six formula units in the unit cell. Its asymmetric unit contains one La and three F sites. Among them, each La^3+^ ion has 11 F^−^ nearest-neighbors arranged into an irregular polyhedron, one F^−^ ion (F1) is tetrahedrally coordinated to La^3+^, and the other F^−^ ions (F2 and F3) are triangularly coordinated to La^3+^ ions. The crystal structure can be considered as a hexagonal closed-packed lanthanum sublattice, with fluorine ions occupying interstitial positions.

It is worth noting that, in previous work [43], Pei et al. reported the preparation of ceria-based compounds with additions of La and F (CLF compounds) by using industrial-grade fluorinated lanthanum–cerium carbonate as a precursor via a facile calcination method. It was found that the compounds consist of three phases: CeO_2_, LaOF, and LaF_3_, of which LaOF has a similar polishing ability to CeO_2_, while LaF_3_ is a low-hardness phase that is easily crushed during polishing, which lowers the MRR and shortens the useful life of the polishing powder. Reasonable content of LaOF and a few LaF_3_ are necessary to prepare CLF compound powders with high polishing performance. The lowest surface roughness (Ra) was achieved when the phase ratio of LaOF was around 10%. The lanthanum–cerium-based abrasive used in this study is a mixture of CeO_2_ (~81%), LaOF (~11%), and LaF_3_ (~8%) (see Table 1), in which the proportion of the LaOF phase is close to the optimum one reported by Pei et al. Therefore, a low surface roughness and good surface quality of polished glass can be expected.

### 3.5. Effect of SHMP on the Surface Ce^3+^ Concentration

**Figure 8:**
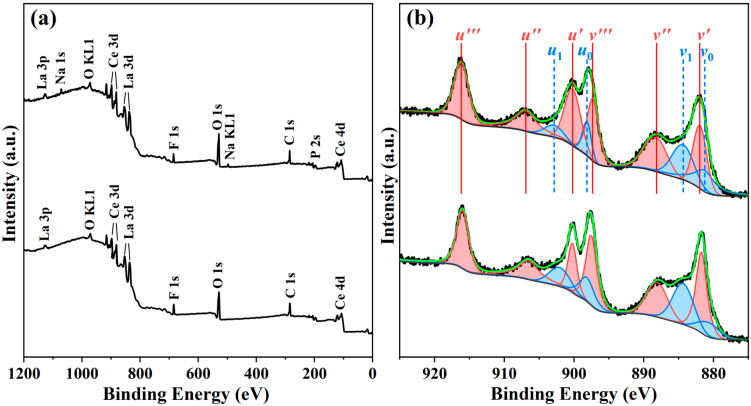
Survey (**a**) and core-level spectra of Ce 3d (**b**) for the 5% lanthanum–cerium-based polishing slurry (bottom) and the 5% lanthanum–cerium-based polishing slurry + 0.5% SHMP (top).

Although cerium in CeO_2_ theoretically exists in the Ce^4+^ oxidation state, due to oxygen deficiency, Ce^3+^ and Ce^4+^ ions may coexist on the surface of CeO_2_. To analyze the differences in surface chemical components of the abrasives with and without SHMP treatment, the polishing slurries consisting of 5% lanthanum–cerium-based abrasives doped with 0.5% SHMP and without SHMP were centrifuged and dried, and wide-survey XPS spectra of the obtained solid particles were recorded in the range of 1200–0 eV, as shown in Figure 8a. The spectra displayed the characteristic photoelectron peaks of all expected elements (La, Ce, O, and F) and the O KLL Auger electron peak. For the sample doped with SHMP, Na 1s, Na KLL Auger, and P 2s signals were also observed. No other impurities were identified in either sample, except for carbon, which may result from the adventitious hydrocarbon from the XPS instrument. The C 1s peak at 284.6 eV was used as a standard to adjust other peaks.

To further obtain the surface Ce^3+^ concentration, the high-resolution Ce 3d core level spectrum was recorded, as shown in Figure 8b. It is generally accepted that the Ce 3d spectrum is complicated due to the presence of two different cerium oxidation states, the hybridization of the O 2p valence band with the Ce 4f level, and spin-orbit coupling [44,45,46]. To identify the specific oxidation state of cerium ions, the spectrum was deconvoluted using the mixed Gaussian–Lorentzian function after eliminating the background, and the results are also provided in Figure 8b.

From this figure, ten deconvolution peaks could be identified, which were labeled as *u*_0_, *u*_1_, *u*′, *u*″, and *u*′′′ for the 3d_3/2_, and *v*_0_, *v*_1_, *v*′, *v*″, and *v*′′′ for the 3d_5/2_ ionizations, respectively [36]. Among these peaks, two doublets, (*u*_0_, *v*_0_) and (*u*_1_, *v*_1_), belong to two different final states of Ce^3+^ in CeO_2_ due to the emission from the spin-orbit split 3d_3/2_ and 3d_5/2_ core levels, while the remaining three doublets, (*u*′, *v*′), (*u*″, *v*″), and (*u*′′′, *v*′′′), are attributed to different final states of Ce^4+^. The concentration of Ce^3+^ ions on the surface of the abrasive can be calculated by dividing the peak area corresponding to Ce^3+^ by the total peak area of Ce 3d according to the following formula (Equation (3), [24,36,47]):

(3) $$[{\mathrm{Ce}}^{3+}]=\frac{A(u_{0})+{A(v}_{0})+A(u_{1})+A(v_{1})}{{A(u}_{0})+{A(v}_{0})+A(u_{1})+A(v_{1})+{A(u}^{\prime })+A(v^{\prime }){+A(u}^{\prime \prime })+{A(v}^{\prime \prime })+{A(u}^{\prime \prime \prime })+A(v^{\prime \prime \prime })}$$

where [Ce^3+^] and A stand for the surface Ce^3+^ concentration and the area of each fitting peak, respectively. The calculated results are summarized in Table 2, which indicates that the Ce^3+^ content has decreased slightly from 31.01% to 28.27% with the SHMP concentration increasing from 0% to 0.5%.

Many studies have shown that Ce^3+^ on the surface of the CeO_2_ abrasive plays an important role in the CMP process, and it is more easily adsorbed on the material surface and removed under the action of mechanical force than Ce^4+^ [36,48,49]. Based on the “chemical tooth” model proposed by Cook [50], a series of bonding reactions occur at the glass–abrasive interface, as described in Equations (4)–(6). It can be seen that the SiO_2_ surface is expected to be terminated with Si–OH due to the hydrolysis reaction [Equation (4)]. Ce–OH groups are also formed when CeO_2_ abrasives are dispersed in water [Equation (5)]. Ce^3+^ (instead of Ce^4+^) ions act as active sites for H_2_O dissociation and help to form Ce–OH groups on the surface of CeO_2_. Silicate ions can adsorb onto the CeO_2_ surface by interacting with the −OH groups to form the Ce–O–Si bonds [Equation (6)], which are stronger than Si–O–Si bonds, thereby realizing the removal of the glass surface layer [49]. More Ce^3+^ on the surface of the abrasive in the slurry is beneficial for the CMP process, as it creates more available active sites at the particle/workpiece interface, which is more favorable for the Ce–OH hydration layer to be formed, thereby accelerating the chemical reaction between the ceria abrasive and glass substrate. In the present case, the surface Ce^3+^ concentration slightly decreased with the addition of SHMP. This means that the improvement of polishing performance by adding SHMP is mainly because SHMP can help disperse CeO_2_ particles into the aqueous medium and improve the hydrophilicity of the particle surface, which will increase the number of effective polishing particles and the contact area between abrasive and glass, thereby enhancing the mechanical rather than chemical effect in the glass CMP process.

≡Si−O−Si≡ + H−OH ↔ ≡Si−OH + HO−Si≡ (4)

=Ce−O−Ce= + H_2_O ↔ =Ce−OH + HO−Ce= (5)

=Ce−OH + HO−Si≡ ↔ =Ce−O−Si≡ + H_2_O (6)

### 3.6. Effect of SHMP on Polishing Performance

**Figure 9:**
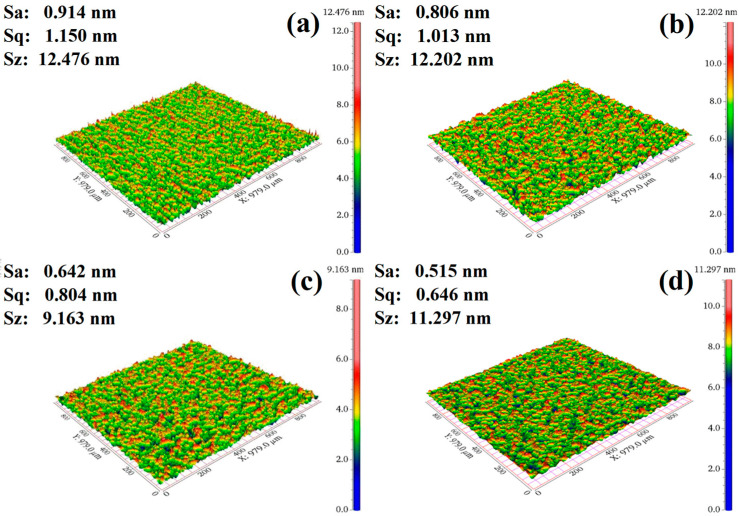
Surface profiles of glass substrates polished by: (**a**) 5% lanthanum–cerium-based polishing slurry; (**b**) 5% lanthanum–cerium-based polishing slurry + 0.5% SHMP (pH = 6.69); (**c**) 5% lanthanum–cerium-based polishing slurry + 0.5% SHMP (pH = 6); (**d**) 5% lanthanum–cerium-based polishing slurry + 0.5% SHMP (pH = 11).

Surface quality after polishing is a crucial index for assessing the CMP effect. A series of polishing tests was carried out on the K9 glass workpieces using the prepared polishing solution and the NF-300 system for 20 min. Subsequently, the polished workpieces were cleaned with deionized water, dried, and evaluated for scratches on the glass surface employing a ZYGO 3D surface profiler, where the surface roughness Ra was used to characterize the surface quality, which was automatically obtained by the software of the ZYGO NewView 5000^TM^ instrument. The smaller the Ra value is, the higher is the surface smoothness, and therefore, the better is the polishing performance. Figure 9 shows the typical three-dimensional surface profiles of glass substrates polished by 5% lanthanum–cerium-based slurry without SHMP and slurry doped with 0.5% SHMP at different pH values. It is obvious that when a 5% lanthanum–cerium-based slurry was used for polishing the glass surface, the surface roughness Ra of the polished surface was relatively large, at 0.914 nm. In this case, the polished surfaces of glass substrates exhibited unevenness, with visible scratches, pitting, or severe contamination observed. With the addition of 0.5% SHMP to the 5% lanthanum–cerium-based slurry and the adjustment of the solution pH value from 6.69 to 6, and then to 11, the surface roughness Ra continuously decreased from 0.806 to 0.642, and finally to 0.515 nm. Obviously, at pH = 6 and 11, the polishing solution composed of 5% lanthanum–cerium-based abrasive doped with 0.5% SHMP had the second lowest and lowest surface roughness Ra, respectively. From the zeta potential (ZP) measurements, it was found that this slurry had two local maximum absolute values of ZP (−48.49 and −51.99 mV at pH = 6 and 11, respectively, as shown in Figure 5). This means that at these two pH values, the electrostatic repulsion between particles reaches a local maximum, and the suspension is the most stable. Therefore, the hard damage caused by abrasive agglomeration will be greatly reduced, and the surface roughness of the K9 polished glass will be significantly decreased. The above results indicated that introducing a certain amount of SHMP into lanthanum–cerium-based abrasives and adjusting the appropriate pH value of the polishing solution can improve the dispersion stability of the slurry and reduce large polishing particles. This makes the force between the abrasive and the glass substrate more uniform, thus effectively reducing surface roughness and improving the surface quality of the K9 polished glass.

Note that many ionic and non-ionic organic surfactants have been used to adjust the composition of polishing slurries [51,52]. They usually have large molecular volumes and low charge densities and can significantly improve the surface coating effect. However, they are generally expensive, and the cost-effectiveness of improving surface electrical properties is not high. In addition, coating excessive organic molecules on the surface of particles can reduce the friction coefficient between the particle surface and the polished workpiece surface, which is not conducive to polishing [53]. Inorganic dispersants, such as SHMP, can overcome these drawbacks. The latter are relatively inexpensive and beneficial for large-scale use in industrial production. More importantly, they have a strong affinity to inorganic particles, and can significantly increase the absolute value of the zeta potential on the particle surface, thereby improving the CMP effect [54]. However, compared to many organic polymer dispersants, SHMP has a relatively small molecular weight, which makes the steric hindrance effect not strong, and its dispersion effect is not satisfactory for some slurries. Furthermore, SHMP readily reacts with Ca^2+^ and Mg^2+^, forming complexes ([M_2_(PO_3_)_6_]^2−^ (where M = Ca or Mg)), which are subsequently adsorbed onto the surface of abrasive particles [55]. Therefore, when used as a slurry dispersant for polishing Ca- and Mg-containing glasses, the white spots of the [M_2_(PO_3_)_6_]^2−^ complexes often appear on the surface of the polished glass.

It is also worth noting that integrated circuit (IC) manufacturing requires exceptionally flat wafer surfaces. Compared to traditional chemical vapor deposition (CVD) and etching techniques, CMP technology has many advantages, such as low cost, high yield, and the capability of achieving global planarization. It has been extensively applied in interlevel dielectric (ILD), shallow trench isolation (STI), damascene metallization, high-k metal gate (HKMG) structures, and three-dimensional logic and memory devices [7]. The rapid development of CMP technology has increased the demand for CMP equipment and created a broad market for consumables (slurries, pads, etc.). The lanthanum–cerium-based abrasive reported in this work may have higher polishing efficiency than SiO_2_ and cause less damage to the silica film than other abrasives such as diamond and alumina [1,10]. It is believed that this polishing slurry may find potential applications in manufacturing optical devices and integrated circuits.

## 4. Conclusions

In this study, SHMP was used as a potential dispersant to improve the dispersion stability of the lanthanum–cerium-based slurry. The liquid contact angle and turbidity tests revealed that the optimal amount of SHMP added to the 5 wt.% lanthanum–cerium-based slurry for achieving the best dispersion effect was about 0.5 wt.%. The obtained polishing solution had a high turbidity of 2715 NTU and a low wetting angle of 45°. The zeta potential (ZP) measurements indicated that this optimal slurry exhibited two local maximum absolute values of ZP (−48.49 and −51.99 mV at pH = 6 and 11, respectively). After polishing with this slurry at pH = 6 and 11, the surface quality of glass substrates was significantly improved, and they showed surface roughness (Ra) values of 0.642 and 0.515 nm (scanning area was 979 μm × 979 μm), respectively. SEM images showed that the proper addition of SHMP resulted in a more uniform distribution of abrasive particles. However, excessive SHMP could cause particles to re-agglomerate due to bridging flocculation, which coincides with the variation trend in turbidity and wetting angle of the slurry. XRD analysis confirmed the coexistence of CeO_2_, LaOF, and LaF_3_ in the abrasive, and the addition of SHMP did not introduce a secondary phase, except for a slight change in phase fraction. XPS studies indicated that the surface Ce^3+^ concentration slightly decreased with the addition of SHMP, suggesting that the improvement of polishing performance by adding SHMP is mainly due to the enhancement of the mechanical rather than chemical effect in the glass CMP process. We believe that the dispersion mechanism of SHMP is a combination of electrostatic repulsion and steric hindrance. This lanthanum–cerium-based slurry doped with a certain amount of SHMP may have potential applications in polishing glass and optical lenses.

## Figures and Tables

**Table 1:** Structural parameters and phase fractions extracted from Rietveld refinements for lanthanum–cerium-based abrasives without and with the addition of SHMP.

Sample		Phase	Space Group	Lattice Parameters			Z	Atomic Coordinates	Phase
				*a* (Å)	*c* (Å)	V (Å^3^)			Fraction
5% La–Ce-based polishing slurry	R_p_ = 4.29%	CeO_2_	*Fm*-3*m*	5.4607(6)		162.83(5)	4	Ce1: 4a (0, 0, 0) O1: 8c (1/4, 1/4, 1/4)	80.8(4)%
	R_wp_ = 5.96%	LaOF	*P*4*/nmm*	4.1024(9)	5.858(2)	98.59(6)	2	La1: 2c (1/2, 0, 0.25(4)) F1: 2a (0, 0, 0) O1: 2b (0, 0, 1/2)	10.6(3)%
	GOF = 3.13	LaF_3_	*P*-3*c*1	7.1859(9)	7.3541(10)	328.87(10)	6	La1: 6f (0.653(2), 0, 1/4) F1: 12g (0.3647, 0.0532, 0.081) F2: 4d (1/3, 2/3, 0.187) F3: 2a (0, 0, 1/4)	8.6(2)%
5% La–Ce-based polishing slurry + 0.5% SHMP	R_p_ = 4.82%	CeO_2_	*Fm*-3*m*	5.4609(6)		162.85(6)	4	Ce1: 4a (0, 0, 0) O1: 8c (1/4, 1/4, 1/4)	81.3(4)%
	R_wp_ = 6.82%	LaOF	*P*4/*nmm*	4.1012(8)	5.861(2)	98.58(5)	2	La1: 2c (1/2, 0, 0.25(2)) F1: 2a (0, 0, 0) O1: 2b (0, 0, 1/2)	10.6(3)%
	GOF = 3.91	LaF_3_	*P*-3*c*1	7.1865(10)	7.3535(11)	328.90(10)	6	La1: 6f (0.655(3), 0, 1/4) F1: 12g (0.3647, 0.0532, 0.081) F2: 4d (1/3, 2/3, 0.187) F3: 2a (0, 0, 1/4)	8.0(2)%

**Table 2:** XPS binding energy and peak area of individual peak of Ce 3d for the 5% lanthanum–cerium-based slurries treated with 0.5% SHMP and without SHMP.

		Ce 3d_5/2_					Ce 3d_3/2_					
Peak Assignment		$v_{0}$	$v^{\prime }$	$v_{1}$	$v^{\prime \prime }$	$v^{\prime \prime \prime }$	$u_{0}$	$u^{\prime }$	$u_{1}$	$u^{\prime \prime }$	$u^{\prime \prime \prime }$	
		Ce^4+^	Ce^3+^	Ce^3+^	Ce^4+^	Ce^4+^	Ce^3+^	Ce^4+^	Ce^3+^	Ce^4+^	Ce^4+^	
Slurry without SHMP	Binding energy (eV)	880.71	881.73	884.37	888.07	897.51	898.17	900.24	902.07	906.5	915.96	[Ce^3+^] (%)
	Peak area (%)	4.55	16.16	14.51	12.59	12.70	6.01	8.12	5.94	8.39	11.03	31.01
Slurry treated with SHMP	Binding energy (eV)	881.26	881.97	884.34	888.16	897.33	898.14	900.21	902.83	906.91	916.17	[Ce^3+^] (%)
	Peak area (%)	9.19	12.43	11.13	11.53	13.26	4.46	13.46	3.49	8.89	12.16	28.27

## Data Availability

Data are contained within the article.
